# Duplication and parallel evolution of the pancreatic ribonuclease gene (*RNASE1*) in folivorous non-colobine primates, the howler monkeys (*Alouatta* spp.)

**DOI:** 10.1038/s41598-019-56941-7

**Published:** 2019-12-30

**Authors:** Mareike C. Janiak, Andrew S. Burrell, Joseph D. Orkin, Todd R. Disotell

**Affiliations:** 1 Department of Anthropology, Rutgers University, New Brunswick, NJ USA; 2 Center for Human Evolutionary Studies, Rutgers University, New Brunswick, NJ USA; 3 Department of Anthropology & Archaeology, University of Calgary, Calgary, AB Canada; 4 Alberta Children’s Hospital Research Institute, University of Calgary, Calgary, AB Canada; 5 Department of Anthropology, New York University, New York, NY USA; 6 Institut de Biologia Evolutiva, CSIC-Universitat Pompeu Fabra, Barcelona, Spain; 7 Department of Anthropology, University of Massachusetts, Amherst, MA USA

**Keywords:** Biological anthropology, Evolutionary genetics, Molecular ecology, Evolutionary ecology

## Abstract

In foregut-fermenting mammals (e.g., colobine monkeys, artiodactyl ruminants) the enzymes pancreatic ribonuclease (*RNASE1*) and lysozyme C (*LYZ*), originally involved in immune defense, have evolved new digestive functions. Howler monkeys are folivorous non-colobine primates that lack the multi-chambered stomachs of colobines and instead digest leaves using fermentation in the caeco-colic region. We present data on the *RNASE1* and *LYZ* genes of four species of howler monkey (*Alouatta* spp.). We find that howler monkey *LYZ* is conserved and does not share the substitutions found in colobine and cow sequences, whereas *RNASE1* was duplicated in the common ancestor of *A. palliata*, *A. seniculus*, *A. sara*, and *A. pigra*. While the parent gene (*RNASE1*) is conserved, the daughter gene (*RNASE1B*) has multiple amino acid substitutions that are parallel to those found in *RNASE1B* genes of colobines. The duplicated RNase in *Alouatta* has biochemical changes similar to those in colobines, suggesting a novel, possibly digestive function. These findings suggest that pancreatic ribonuclease has, in parallel, evolved a new role for digesting the products of microbial fermentation in both foregut- and hindgut-fermenting folivorous primates. This may be a vital digestive enzyme adaptation allowing howler monkeys to survive on leaves during periods of low fruit availability.

## Introduction

Howler monkeys (*Alouatta* spp.) are some of the few New World monkeys with a diet rich in leaves. While the amount of leaves consumed varies greatly between sites and populations^1^, as well as across seasons^2^, on average young and mature leaves make up half or more of the howler monkey diet^3–7^. Howler monkeys manage to persist on such a diet without the sacculated, foregut-fermenting stomachs found in folivorous Old World monkeys, the colobines. However, howler monkeys have a suite of other adaptations for a folivorous diet.

Adaptations for a leaf-rich diet include behavioral strategies, such as minimizing energy expenditure^2,8,9^ and feeding preferentially on young leaves^6,10,11^ or leaves with a higher protein to fiber ratio^5^, which are less tough and may be easier to digest^12^. Howler monkeys are unique among platyrrhines in having routine trichromacy, a trait that has been proposed as an adaptation for detecting such young leaves^13,14^. Gut and digestive adaptations include very long gut transit times of approx. 20 hours^15,16^ and an enlarged caecum and colon^17^.

These gut adaptations are important for folivorous mammals because the cell walls of plants are made of structural carbohydrates, cellulose and hemicellulose, that cannot be digested by the enzymes produced by vertebrates^18^. Instead, leaf-eating mammals rely on microbial fermentation to break down plant material in the forestomach (foregut fermenters, like colobines) or in the large intestine (“hindgut” or caeco-colic fermenters)^18–20^. The enlarged caecum and colon in howlers are important sites for microbial fermentation and are also enlarged in other folivorous or herbivorous caeco-colic fermenters, such as horses and elephants^21^. During microbial fermentation, the plant material is broken down by symbiotic bacteria found in the host’s gut. This process releases volatile fatty acids, which are easily absorbed^22^ but the bacteria themselves are also digested by the host and are an important source of nitrogen^22,23^. Both ruminant artiodactyls and colobine monkeys have convergent digestive enzyme adaptations for the digestion of fermenting gut bacteria, but we do not know whether howler monkeys share any of these adaptations. Endogenous digestive enzymes are a crucial component of an animal’s digestive system and include important dietary adaptations^24^, such as the enzymes lysozyme and pancreatic ribonuclease.

### RNase and Lysozyme Evolution in Primates and Ruminants

The enzyme lysozyme is found in many vertebrates and invertebrates where it has an immunological function^25^. In cows and colobines this enzyme exhibits parallel amino acid changes that allow lysozyme to function at a much lower pH, an adaptation for bacteriolytic activity in the acidic stomach fluid^26,27^. Similarly, the pancreatic ribonuclease enzyme (RNase1), which has an original function in pathogen defense^28^, has acquired a new digestive function in both colobines and ruminant artiodactyls^23,29–31^. In this case, the *RNASE1* gene underwent one or more duplications, and the duplicated gene(s) (*RNASE1B* and *RNASE1C*, or alternatively *RNASE1β* and *RNASE1γ*) evolved a new function in digesting the nucleic acids of fermenting microbes found in the digestive system. These duplications and subsequent functional changes evolved independently in artiodactyl ruminants and colobines and, indeed, via different amino acid substitutions^30^. They may have also evolved separately in African and Asian colobines^31,32^, although this has been questioned by some studies^33,34^ and the evolutionary history of *RNASE1* duplications in colobines has not yet been completely resolved.

Significantly, the duplicated RNases of both ruminants and colobines have a set of different, taxon-specific amino acid changes leading to convergent changes of the isoelectric point (*pI*), optimal pH, and charge of the resulting protein. The digestive RNases (RNase1B and RNase1C in colobines, pancreatic RNase1 in bovines) exhibit a lower *pI*, a lower optimal pH, and a decreased charge at pH 7.0^30,31^ compared to the ancestral RNase1. Previous research has shown that the charge of pancreatic ribonuclease affects the enzyme’s ability to degrade double-stranded RNA^35^. A high *pI* and positive charge is indicative of a ribonuclease that is involved in defense against pathogens, while a decrease in *pI* and charge suggest a novel function for the enzyme^36,37^, as shown in the case of cows and colobines^30^.

### RNase and Lysozyme Evolution in Other Mammals

Duplications of the *RNASE1* gene have also been found in other mammal groups. In rodents, both rats (*Rattus* spp.) and guinea pigs (*Cavia* spp.) independently evolved two duplicated genes, *RNASE1B* and *RNASE1C*, from the ancestral *RNASE1* gene^37–39^. Whether these duplicated genes remain involved in immune function or have acquired novel, possibly digestive, functions has not been determined. Lang and colleagues (2017) suggest that the high *pI* (9.66) of rat RNase1B and its expression in the spleen point to retention of the original immunological function, while rat RNase1C (*pI* = 7.71) may have acquired a novel function^39^. Similar to cows and colobines, the duplicated ribonuclease in guinea pigs has a lower *pI* and decreased charge and is less effective at degrading double-stranded RNA than the ancestral enzyme, suggesting a novel and possibly digestive function^35,40^. Like howler monkeys, guinea pigs are herbivorous animals with caeco-colic rather than foregut fermentation. This suggests that ribonuclease could have a function in the digestion of microbial fermentation products, regardless of the presence of a foregut.

*RNASE1* also underwent multiple duplications independently in two families of bats, the Vespertilionidae and Molossidae, but the duplicate proteins have high isoelectric points suggesting an immunological rather than dietary function^41^. Seven *RNASE1* genes were found in *Myotis lucifugus*, an insectivorous Vesper bat^37^. The authors propose that this may be an immunological adaptation, as the communal roosting behavior of these bats potentially increases their exposure to pathogens and RNase1 may improve their resilience to them^37^. In the superfamily Musteloidea, a group that includes red pandas, weasels, raccoons, and skunks, *RNASE1* was duplicated independently in four families but the functional significance of these duplicates is not yet clear^36^. As summarized here, *RNASE1* genes have an interesting history of duplications and functional diversification in mammals, including for novel dietary functions in herbivorous foregut and caeco-colic fermenters.

### Present Study

The *RNASE1* gene(s) of folivorous foregut-fermenting primates (colobines) and many non-folivorous primates are now well characterized^31–33^, but it is not clear whether folivorous caeco-colic fermenting primates, such as howler monkeys, share the digestive enzyme adaptations of colobines. In this study, we therefore investigated both the *RNASE1* and *LYZ* genes in four howler monkey species – the mantled howler (*Alouatta palliata*), the Venezuelan red howler (*A. seniculus*), Guatemalan black howler (*A. pigra*), and the Bolivian red howler (*A. sara*) – a group of leaf-eating primates with caeco-colic fermentation. We used a 10X Chromium draft genome assembly for *Alouatta palliata* and conducted amplicon sequencing of the *RNASE1* gene for all four howler monkey species. We hypothesize that howler monkey RNase1 and lysozyme exhibit similar changes in charge and isoelectric point as the proteins in colobines, as adaptations for a folivorous diet. To find the predicted shared amino acid changes, we assembled a comparative dataset of *RNASE1* and *LYZ* gene sequences across primates and translated and aligned the coding sequences. To better understand the evolutionary history and selective pressures acting on *RNASE1*, we tested for positive/purifying selection and reconstructed the inferred ancestral gene sequences. Finally, the *pI* and charge at pH 7.0 were calculated for both extant and inferred ancestral sequences, in order to identify the predicted shared biochemical properties of the proteins in different species.

## Results

### Genome mining and sequencing

Our *Alouatta palliata* draft genome assembly is 2.51 Gb with a contig N50 of 58.2 kb, a scaffold N50 of 3.47 Mb, and an effective read depth of 38X (Burrell *et al*., in prep; Janiak *et al*., in prep). From these reads, we were able to identify sequences in the *Alouatta palliata* draft genome putatively orthologous to the query *LYZ* and *RNASE1* gene sequences.

**Figure 1:**
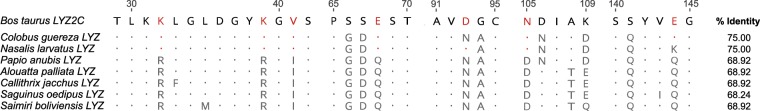
Primate *LYZ* protein sequences aligned to cow (*Bos taurus*) reference sequence. Parallel amino acid changes between cow and colobines are highlighted. Percent identity to the cow reference sequence is indicated on the right.

The *LYZ* nucleotide sequence of *A. palliata* was 96.42% and 96.20% identical to that of *Saimiri boliviensis* and *Callithrix jacchus*, respectively. The *LYZ* amino acid sequence was also very similar to those of other platyrrhines, being 91.22–91.89% identical. A protein alignment of the lysozyme C sequences from *A. palliata*, *S. boliviensis*, *C. jacchus*, *Saguinus oedipus*, *Papio anubis*, *Colobus guereza*, *Nasalis larvatus*, and the cow (*Bos taurus*) is shown in Fig. 1. While colobines and cows have a number of parallel amino acid changes, these substitutions are not found in howler monkey lysozyme C (Fig. 1). Pairwise distances of lysozyme C amino acid sequences are shown in Supplemental Table S3. As found in previous studies^26^, the two colobines (*C. guereza* and *N. larvatus*) have overall greater sequence similarity with the cow (111/148 amino acids, 75%) than another closely related catarrhine, *Papio anubis*, has with the cow (102/148 amino acids, 68.92%). The howler monkey, on the other hand, does not share the parallel changes found in the lysozyme sequences of colobines and cows and its sequence identity with these groups is comparable to those of other platyrrhines (Fig. 1, Suppl. Table S3). Like the other platyrrhines, the howler monkey shares 124–125/148 amino acids (83.78–84.46%) with the *LYZ* sequence of colobines and 102/148 amino acids (68.92%) with the *LYZ* sequence in cows.

The *RNASE1* BLASTN search of the howler monkey Supernova 10X genome pseudohap assembly initially returned no hits that matched the *Callithrix jacchus RNASE1* query. Because *RNASE1* is conserved in all other primates, it is implausible that this gene was lost in *Alouatta*. We, therefore, repeated our BLASTN search on the raw Supernova assembly output which contains every edge in the assembly and does not flatten bubbles^42^. The BLASTN search of the raw Supernova assembly output produced a total of twelve significant alignments ranging in similarity from 92–98%. However, none of the hits covered the entire query sequence and parts of the query produced multiple non-identical hits.

### Amplicon sequencing

**Figure 2:**
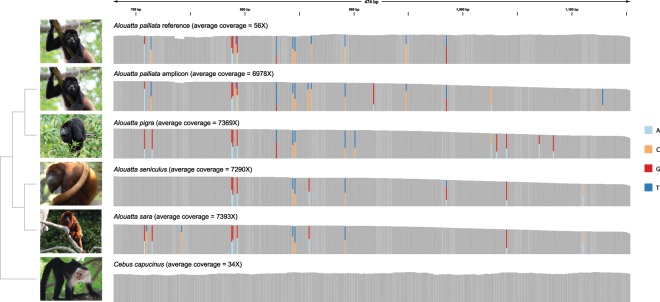
Coverage of reads (duplicates removed) mapped against *RNASE1* coding region. Variable sites are colored by proportion of reads with each base at that site. *Cebus capucinus imitator* is a non-folivorous platyrrhine that was included as a control. Reads mapping to *RNASE1* in *C. capucinus* do not indicate the presence of variable sites. Images by Simon Pierre Barrette (*Alouatta palliata*), Dave Johnson (*A. pigra*), Alessandro Catenazzi (*A. seniculus*), Raul Ignacio (*A. sara*), and Steven G. Johnson (*Cebus capucinus*), all via Wikimedia Commons.

Sequencing of the *RNASE1* amplicons on an Illumina MiSeq produced an average of 1,812,320 reads per sample (SD = 100,204 reads), of which an average of 65.86% mapped to the reference (range = 44.07–83.42%) (Suppl. Fig. S7). Average coverage of the *RNASE1* coding region ranged from 6978–7393X (Fig. 2) for reads generated from amplicon sequencing. The average coverage of the *RNASE1* coding region for the reads generated for the *A. palliata* genome assembly was 56X, while reads from whole genome sequencing of *Cebus capucinus imitator* had an average coverage of 34X (Fig. 2).

### Assembly of RNASE1

Read mapping, followed by variant calling revealed 10–19 variable sites above a PHRED-scaled quality threshold of 20 in the coding region of *RNASE1* (Suppl. Table S4). Six of these variants were shared between all four species. Haplotype calling and assembly of the *RNASE1* coding region yielded three distinct haplotypes for each of the *Alouatta* species and a single haplotype for *Cebus capucinus imitator* (Fig. 2). The two most similar haplotypes were 98.28–99.57% identical, while the two most divergent haplotypes were 94.18–97.20% identical in each species (Suppl. Table S5).To exclude the possibility that these sequences were of another, closely related gene, we conducted BLAST searches with all haplotype sequences as queries against publicly-available platyrrhine genomes (*n* = 4). All searches only returned hits to *RNASE1* and no other platyrrhine genomes investigated here showed evidence of a second *RNASE1*-like gene. Therefore, it is most likely that the additional *RNASE1*-like sequences found in the howler monkey genome represent a duplication of the ancestral *RNASE1* gene. The observed read depths and haplotype frequencies (Suppl. Table S4) suggest that *Alouatta* species have two *RNASE1* genes and that the third haplotype represents small polymorphisms within one or both of these genes. We refer to these duplicated sequences as *RNASE1B*.

### Sequence analyses

Primate *RNASE1* genes generally appear to be conserved and lack premature stop codons in all species included here (*n* = 29). The overall amino acid sequence divergence across *RNASE1* in these species was low (mean pairwise identity = 88.63%, SD = 4.78%). When including the duplicated *RNASE1* genes found in colobines and *Alouatta*, overall divergence only increased slightly (mean pairwise identity = 87.20%, SD = 4.86%).

**Figure 3:**
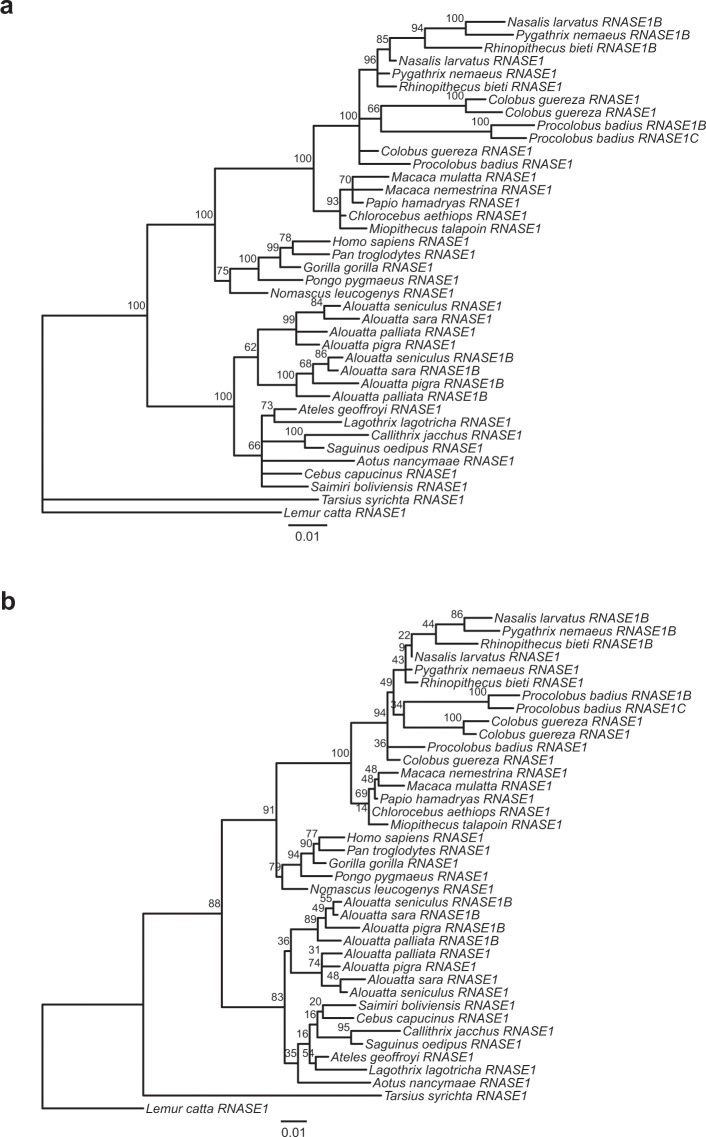
Phylogenies of primates based on coding sequences (474 bp) of *RNASE1* and duplications. Trees were built using a (**a**) Bayesian approach with MrBayes^60^ and a b) maximum-likelihood method with PHYML^61^. Branch labels indicate (**a**) the posterior probability in percent and (**b**) bootstrap support in percent (1000 replicates). Scale bars indicate rate of substitutions per site.

Trees built from the coding region of *RNASE1* failed to accurately resolve the phylogenetic relationships of all primate species we examined (Fig. 3). Different programs (MrBayes, PHYML) and approaches (neighbor-joining, maximum likelihood) gave different topologies and did not resolve the history of *RNASE1* duplications in colobines with confidence. This is likely due to the short size of (471 bp) and overall conservation of *RNASE1*. However, all approaches supported a scenario in which *RNASE1* was duplicated once in the common ancestor of *Alouatta* species. Sequence information for non-coding regions of *RNASE1* was not available for all species in our sample, so it was not possible to use a longer sequence to construct a phylogenetic tree.

CODEML provided evidence that there was an increase in the underlying mutation rate of the ancestral *RNASE1B* branch (H1: ΔLRT = 5.77, *p* = 0.016; Table 1) and that there has been positive selection for functional divergence in *RNASE1B* following the duplication event (H2: ΔLRT = 6.47, *p* = 0.011; Table 1). This is further supported by the dN/dS ratios in model H2, which were above one for the duplicated branches (*ω* = 1.229) but below one on the background branches (*ω* = 0.287). Note that the value for omega for the foreground branches in model H1 (*ω* = 999.0) is due to dS = 0, which makes it unreliable (Table 1). However, the LRT is unaffected by this, so we are basing our conclusions for model H1 only on the comparisons with the null model. The branch-site model did not support the hypothesis that any sites along the howler monkey *RNASE1B* genes are under positive selection, as the alternative model did not have a significantly better fit than the null model (ΔLRT = 3.41, *p* = 0.065; Table 1).

**Table 1:** Results of CODEML analyses for primate *RNASE1* and *Alouatta* duplicated sequences (n = 33).

Analysis	Model	ω (dN/dS)	lnL	LRT	*p*	Test
Branch	H0	0.313	−2159.77			Null model, one *ω* for all branches
	H1	Background = 0.301	−2156.89	5.77	0.016	Higher *ω* on branch leading to duplicated genes?
		Foreground = 999.0				
	H2	Background = 0.287	−2156.54	6.47	0.011	Higher *ω* on duplicated branches?
		Foreground = 1.229				
Branch-site	Null		−1948.78			Are there positively selected sites along *RNASE1B*?
	Alternative		−1947.08	3.41	0.065	

### Protein properties and ancestral sequence reconstruction

**Figure 4:**
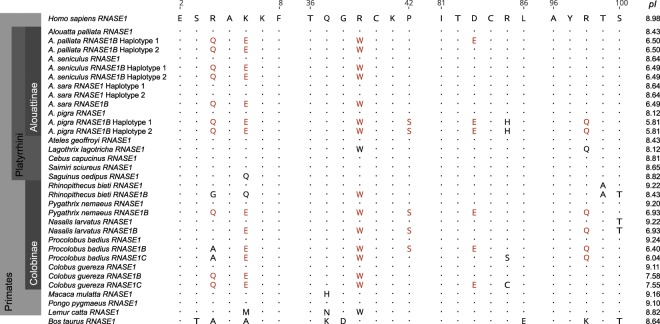
Primate *RNASE1*, *RNASE1B*, and *RNASE1C* sequences aligned to human (*Homo sapiens*) reference sequence. Parallel amino acid changes between duplicated genes (*RNASE1B* and *RNASE1C*) in *Alouatta palliata* and colobines are highlighted in red. Numbering is in relation to the start of the mature peptide sequence and consistent with numbering used in previous studies^30,31^. Isoelectric points (*pI*) shown on the right. *PI* calculated with ExPASy Compute pI tool.

**Figure 5:**
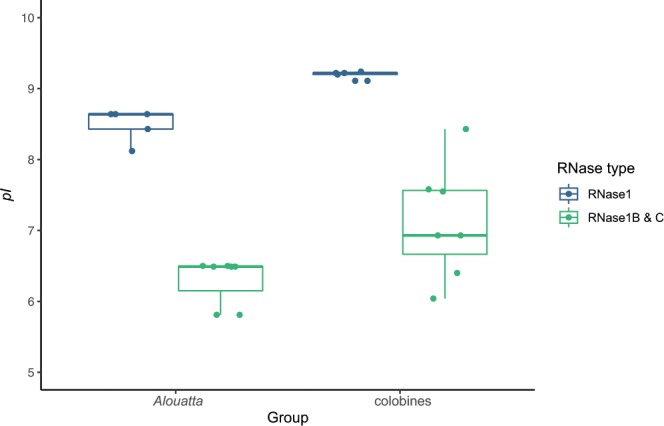
Computed isoelectric points (*pI*) of RNase1 and the duplicated proteins RNase1B and RNase1C in colobines and *Alouatta* spp.

Aligning all RNase1 protein sequences shows several parallel amino acid changes between the duplicated howler monkey and colobine genes, *RNASE1B* and *RNASE1C* (Fig. 4), but not with the bovine pancreatic *RNASE1* (Suppl. Fig. S8). These include functionally important changes that have been experimentally shown to decrease the enzyme’s activity against double-stranded RNA in colobine monkeys^30,31,43^. The duplicated sequences in all *Alouatta* species have a change from arginine to glutamine at site 4, a change from lysine to glutamic acid at site 6, and a change from arginine to tryptophan at site 39. *A*. *palliata* and *A*. *pigra* share an additional change from aspartic acid to glutamic acid at site 83 with the duplicated colobine proteins. Arginine and lysine are positively charged amino acids, while glutamic acid is negatively charged, so these changes contribute to a change in the charge of the resulting protein. *Alouatta pigra* further shares changes from proline to serine at site 42 and from arginine to glutamine at site 98. All numbering is in relation to the start of the mature peptide sequence and consistent with site numbering used in Zhang (2003)^30^. The isoelectric point (*pI*) of RNase1 and the duplicated RNase1 proteins are shown in Fig. 5. Compared to the high *pI* (9.11–9.24) and higher charge of parent RNase1 proteins, the duplicated proteins in colobines have a lower *pI* (6.04–8.43) (Fig. 5) and a reduced charge at pH 7.0 (Fig. 4). Likewise, the parent howler monkey RNase1 proteins have a higher *pI* (8.12–8.64) and charge (2.9–4.9) than the duplicated howler proteins (*pI* = 5.81–6.50, charge = −2.9 to −0.1) (Figs. 4,5).

**Figure 6:**
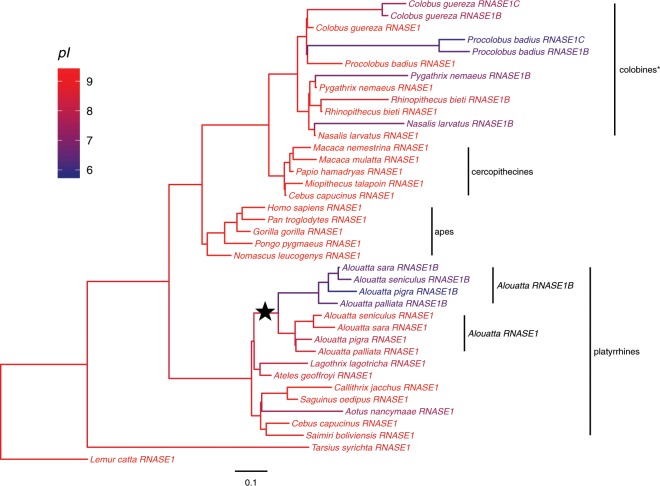
Evolutionary relationships and isoelectric point (*pI*) of the proteins encoded by *RNASE1*, *RNASE1B*, and *RNASE1C* in primates. Branches are colored based on computed *pI* of extant primate protein sequences and reconstructed ancestral protein sequences. The timing of the *RNASE1* duplication in *Alouatta* is indicated by the black star. *The colobine clade as shown here likely does not reflect the true evolutionary history of *RNASE1* and gene duplications. Because the evolutionary history of the *RNASE1* genes in colobines has not been fully resolved, the colobine genes are grouped by species here to illustrate the differences in *pI* between the parent and daughter proteins.

Reconstruction of the ancestral *RNASE1* sequences showed that isoelectric points of RNase1 proteins are consistently high across the primate phylogeny, with the exception of the duplicated proteins in colobines and *Alouatta* (Fig. 6). Another exception is the RNase1 protein of the owl monkey (*Aotus nancymaae*). While we found no evidence of a duplicated gene, the *Aotus RNASE1* sequence has three amino acid changes that are parallel to changes found in the colobine *RNASE1B* sequences (K1G, K6E and R39W). Consistent with these changes, the *pI* of owl monkey RNase1 is lower (7.56) than any other non-duplicated RNase1 in primates, but still higher than howler monkey RNase1B (Fig. 6, Supplemental Table S6). The *RNASE1* sequence of the brown woolly monkey (*Lagothrix lagotricha*) shares two of the amino acid changes found in *RNASE1B* (R39W and R98Q) but the protein does not exhibit a strong decrease in *pI* (Fig. 4, Supplemental Table S6) and there is no evidence of a gene duplication^43^. Other non-folivorous New World primates have conserved RNase1 sequences that do not share any or at most one of these amino acid substitutions (Fig. 4, Suppl. Fig. S8).

## Discussion

In many mammal groups *RNASE1* genes have a history of duplications and functional diversification. This study identified a previously unknown *RNASE1* duplication event in the common ancestor of extant howler monkey (*Alouatta*) species and found amino acid substitutions in the duplicated gene that are parallel to those in duplicated *RNASE1* genes in colobines and consistent with a new function in the digestive system. The howler monkey *LYZ* gene, however, was found to be conserved and not to have evolved changes consistent with a new role as a digestive enzyme.

While all other platyrrhine species studied so far only have one *RNASE1* gene^43^, two different *RNASE1*-like sequences were identified in *Alouatta palliata*, *A. seniculus*, *A. sara*, and *A. pigra*. One sequence (*RNASE1*) retained a high *pI* and positive charge, while the other sequence (*RNASE1B*) had several amino acid changes (Fig. 4) that resulted in a reduction of the proteins’ *pI* and charge (Figs. 4,5). Such changes have also been found in *RNASE1* duplications in Asian and African colobine monkeys and artiodactyl ruminants^30–33^. Colobine RNase1B and RNase1C have up to nine amino acid changes that reduce the enzymes’ effectiveness against double stranded RNA^43^ and howler monkey RNase1B proteins share three (R4Q, K6E, R39W), four (R4Q, K6E, R39W, D83E), or six (R4Q, K6E, R39W, P42S, D83E, R98Q) of these substitutions, depending on the species (Fig. 4). It is therefore likely that the duplicated howler monkey proteins are not as effective against double-stranded RNA as the ancestral protein. Combined with the lowered *pI* and reduced charge, this supports the idea that this *RNASE1* duplication in howler monkeys has diverged in function from the original, immunological role of the ancestral protein^23,35–37,43^. Zhang (2006) had previously calculated the probability of three or more parallel amino acid substitutions arising in two lineages by chance to be between 0.0001 and 0.0026^31^. The more likely explanation is that the parallel substitutions in howler monkey and colobine *RNASE1B/C* arose due to shared selective pressures. Results from the CODEML branch models further support the hypothesis that there was positive selection for functional divergence following the duplication event (Table 1). However, we want to caution that omega values are often elevated following gene duplication^44,45^, potentially due to loss of function and relaxed selection. Thus, relaxed selection following gene duplication rather than positive selection for functional divergence may be an alternate explanation for the results of the CODEML analyses.

In colobines and ruminant artiodactyls the duplicated RNase1 proteins are thought to be adaptations for the digestion of bacteria that ferment leaves in the foregut of these animals, an important source of nitrogen^29,33^. While howler monkeys do not have sacculated forestomachs like colobines and ruminants, they do rely on microbial fermentation, just in the caeco-colic region, rather than forestomach, to break down the foliage they consume^19^. The duplicated RNase1B may thus fill a role similar to the duplicated proteins in colobines and artiodactyl ruminants, by efficiently digesting bacterial RNA in the caeco-colic region, which has a similar pH (6.8) to that reported for the small intestine in colobines (6.0–7.0)^19,22,43^. In a study of fermentative digestion in *Alouatta palliata* the authors found that up to 31% of the monkeys’ daily required energy may come from the digestion of fermentation end products^19^. Although caeco-colic fermentation of leaves has been shown to be slightly less efficient at digesting fiber than foregut fermentation^21,46^, the overall nutritional gains may be comparable if the digestion of fermentation end products is considered. During times of fruit scarcity when the howler monkey diet consists entirely of leaves, the ability to efficiently digest such products may therefore be crucial to their survival^19^ and the RNase1B enzyme may be a key factor ensuring digestive efficiency.

Unlike cows and colobines, howler monkeys did not have parallel amino acid changes in the *LYZ* gene (Fig. 1). Lysozyme is an enzyme found in many vertebrates and invertebrates and is thought to play a role in immune function^25^. In cows and colobines, however, lysozyme exhibits parallel amino acid changes that allow the enzyme to function at a much lower pH, possibly as an adaptation for bacteriolytic activity in the acidic stomach fluid^26,27^. In artiodactyl ruminants the *LYZ* gene underwent multiple duplications and some of the daughter genes acquired a novel digestive function^47^. Interestingly, in the colobines there is only a single *LYZ* gene that was adapted for a digestive function. In non-colobine primates, including howler monkeys, *LYZ* is conserved (Fig. 1)^48^. It may be that the utility of lysozyme as a digestive enzyme is tied to foregut-fermentation, while ribonuclease can be adaptive for microbial fermentation both in the foregut, as well as in the “hindgut.” Some support for this is provided by a study of lysozyme in the only avian species with foregut-fermentation, the hoatzin (*Opisthocomus hoazin*), a leaf-eating bird from South America^49^. Despite arising from a different lysozyme gene family, a lysozyme expressed in the hoatzin stomach has biochemical properties and amino acid substitutions that are parallel to those found in artiodactyl ruminants and colobines^50^. Another possible example of ribonuclease adaptation for a digestive purpose comes from the ancient DNA of the extinct subfossil lemur *Megaladapis* (Perry and colleagues, in prep). Based on studies of dental microwear and dental topography *Megaladapis* was likely folivorous^51,52^ and its *RNASE1* gene shares several amino acid substitutions with the duplicated *RNASE1B* and *RNASE1C* genes of colobines and howler monkeys (Perry and colleagues, in prep). Since no extant lemurs have a colobine-like digestive system^18^, it is most likely that *Megaladapis* relied on caeco-colic fermentation to digest leaves, like howler monkeys.

A limitation of the current study is that data are lacking on where in the howler monkey body *RNASE1* and *RNASE1B* are expressed. Finding that *RNASE1B* is expressed in the howler monkey caecum and/or colon would provide strong evidence that this protein has been repurposed as a digestive enzyme. Future expression studies should therefore be a priority. The role of *RNASE1* in owl monkeys (*Aotus* spp.) likewise deserves additional study. While there is no evidence of a gene duplication, *A. nancymaae RNASE1* shares three amino acid substitutions with colobine *RNASE1B/C* and consequently has a lower *pI* than RNase1 in other species (Fig. 6, Suppl. Table S6). Zhang (2006) has noted that evolution of a digestive role for pancreatic RNase is likely impossible without gene duplication, given the need to retain an RNase with efficient double-stranded RNA degradation activity^31^. Kinetic assays of owl monkey RNase1 are, therefore, a priority for future research. Finally, some populations of the New World primate genus *Brachyteles*^53^ and several lemur species^54^ are also quite folivorous, making them targets for future studies.

## Conclusions

The *RNASE1* gene family has a history of duplications and functional divergence in many mammals, including the colobine primates^30–33,36,37,39,41^. Here we present evidence that *RNASE1* has also been duplicated in the common ancestor of a group of folivorous non-colobine primates, the howler monkeys (*Alouatta* spp.), and that the duplicated gene (*RNASE1B*) has biochemical properties and amino acid substitutions that are parallel to those found in foregut-fermenting primates. This protein may therefore be used for an analogous function in howler monkeys, digesting the products of microbial fermentation in the caeco-colic region, a potentially substantial source of energy and nitrogen^19^. Along with behavioral and morphological adaptations, this duplicated protein may be a crucial digestive enzyme adaptation allowing howler monkeys to survive on a folivorous diet during times of fruit scarcity.

## Methods

### Genome mining and sequencing

To assemble a comparative dataset of pancreatic ribonuclease (*RNASE1*) and lysozyme (*LYZ*) gene, we mined primate genomes and gene sequences available on GenBank, as well as an unpublished draft genome assembly of the mantled howler monkey (*Alouatta palliata*) that we generated using Chromium Genome library preparation (10X Genomics) at New York University’s Langone School of Medicine Genome Technology Center for another project (Burrell *et al*., in prep; Janiak *et al*., in prep). Genomic DNA was already extracted and came from the collection of the Molecular Anthropology Lab at New York University. We size-selected high-molecular weight DNA via a Blue Pippin (Sage Science) for fragments > 50 kb long prior to library prep. The Chromium Genome library was then run on two lanes of an Illumina HiSeq. 2500 with v. 4.0 chemistry. Reads were assembled with the 10X Genomics Supernova pipeline^42^.

The *RNASE1* sequence from the common marmoset (*Callithrix jacchus*) reference genome and the *LYZ* sequence from the black-capped squirrel monkey (*Saimiri boliviensis*) reference genome were used as queries to run BLASTN searches (default search parameters) on the Supernova 10X genome pseudohap and raw assemblies of *Alouatta palliata*. Primate *RNASE1* sequences generated in previous studies^31–33,43^ were downloaded from the National Center for Biotechnology (NCBI, accession numbers in Supplemental Table S1). For a better understanding of the history of *RNASE1* in primates, especially in platyrrhines, the published reference genomes of *Aotus nancymaae*, *Cebus capucinus imitator*, *Microcebus murinus*, and *Tarsius syrichta* were searched for *RNASE1* sequences using BLASTN with the same query and parameters as above. Primate sequences of *LYZ* generated in a previous study^48^ were retrieved from GenBank. The *LYZ* gene sequence found in the *Bos taurus* reference genome differed from the sequence reported in Stewart *et al*. (1987)^26^. The sequence for *Bos taurus LYZC2* was used, because it was most similar and almost identical to the bovine lysozyme sequence reported in Stewart *et al*. (1987). A full list of sequences used in this study and their accession numbers are presented in Supplemental Table S1.

### Amplicon sequencing

In addition to genome sequencing of *A*. *palliata*, we also conducted amplicon sequencing of the *RNASE1* gene region in four howler monkey species (*Alouatta palliata*, *A. seniculus*, *A. pigra, A. sara*). Briefly, we amplified a 1180 bp region surrounding *RNASE1* using conserved PCR primers (Suppl. Table S2) and KAPA HiFi HotStart ReadyMix (thermocycler settings in Suppl. Table S2), gel purified the amplicons (PureLink Quick Gel Extraction Kit, Invitrogen), before shearing them with a Covaris S2 focused-ultrasonicator (settings used: intensity = 2, duty factor = 10%, cycles/burst = 200, time = 45 sec, cycles = 2). The sheared samples were prepped with an NEBNext Ultra II DNA Library Prep Kit (New England Biolabs) and sequenced together on an Illumina MiSeq using a Micro Kit with v2 chemistry. Extracted DNA samples used in this study were from the collection of the Molecular Anthropology Lab at New York University.

### Assembly of RNASE1

Assembly with the 10X Genomics Supernova pipeline was unable resolve the *RNASE1* region in *Alouatta palliata*. We, therefore, attempted to complete a targeted re-assembly of the region of interest from the *A. palliata* Illumina short reads. In order to use reads generated with the 10X Genomics system with standard mapping and assembly tools, it is necessary to remove the 10X linked barcodes from the reads. We removed barcodes with the script process_10xReads.py (https://github.com/ucdavis-bioinformatics/proc10xG). The 10X Genomics short reads (with barcodes removed), the short reads resulting from amplicon sequencing (after demultiplexing and adapter trimming), and short reads from *Cebus capucinus imitator* were mapped to the human *RNASE1* region with bwa mem^55^ (https://github.com/lh3/bwa), followed by sorting and indexing with samtools^56^. After the initial mapping to the human *RNASE1* reference, we identified the consensus sequence of each set of mapped reads and repeated the mapping step with this consensus sequence as a reference.

After mapping, sorting, indexing, and removing duplicates, we called variants and then haplotypes (using the previously called variants as a guide) with freebayes v1.2.0–17-ga78ffc0^57^. We then reviewed and confirmed the short haplotypes identified by freebayes in the Integrative Genomics Viewer (IGV)^58^ and manually assembled the full-length *RNASE1* haplotypes for each species.

### Sequence analyses

Coding regions of all sequences for *RNASE1* (464–474 bp) and *LYZ* (447 bp) were translated using Geneious 9.1.8. Coding regions and translated amino acid sequences were aligned using MAFFT v7^59^ and Geneious 9.1.8 (alignments are available as supplementary materials). The following analyses were only conducted with data for *RNASE1*, because no evidence of duplication or convergence between *Alouatta* and colobines was found for *LYZ*.

Phylogenetic trees were constructed from both nucleotide and protein alignments of *RNASE1* with MrBayes^60^ and PHYML^61^ programs using the Hasegawa-Kishino-Yano (HKY) substitution model with a discrete Gamma distribution (+G). The nucleotide substitution model was chosen based on the Akaike Information Criterion (AIC) statistics calculated by the jModelTest program (http://jmodeltest.org/)^62^. In PHYML, 1000 bootstrap replicates were completed. Because these relatively short sequences did not resolve the primate phylogeny accurately, trees based on the best available primate phylogeny^63^ were used in CODEML analyses and ancestral sequence reconstructions.

We used branch and branch-site models in the program CODEML which is part of the PAML package^64^ to test for positive selection acting on the duplicated *RNASE1* genes in howler monkeys. Branch-specific models (H0, H1, and H2) were used to determine if there is an increase in the underlying mutation rate of the ancestral *RNASE1B* branch (H1) and if there is evidence of positive selection for functional divergence in *RNASE1B* following the duplication event (H2). Model fit was evaluated using likelihood ratio tests (LRT). Variation in the values of *ω* (nonsynonymous/synonymous substitutions, d_n_/d_s_) across sites along the *RNASE1B* genes was evaluated with branch-site models. For these, the duplicated *Alouatta RNASE1* genes (*RNASE1B*) and the ancestral branch leading to them were designated as foreground branches, while all parent *RNASE1* genes were designated as background branches. In this model, *ω* is allowed to vary both between sites and across branches to determine whether any sites are under positive selection in the foreground branches^64^. This alternative model is compared to the null model in which *ω* is fixed at 1 and model fit is evaluated with a LRT.

### Protein properties

To compare the biochemical properties of proteins across species, the isoelectric points (*pI*) of lysozyme, extant RNAse1 and inferred ancestral RNAse1 proteins were calculated with the Compute pI Tool on the ExPASy webserver (http://web.expasy.org/compute_pi/). Charge at pH 7 was calculated with ProteinCalculator v3.4 (http://protcalc.sourceforge.net/).

### Ancestral sequence reconstruction

To better understand the evolutionary history of *RNASE1* in primates, ancestral sequences were reconstructed (n = 38) using FastML (http://fastml.tau.ac.il/)^65^ with a dataset of 39 sequences (shown in Fig. 6). The following running parameters were used: sequence type = codons, model of substitution = yang, use gamma distribution = yes and probability cutoff to prefer ancestral indel over character = 0.5. Reconstructed ancestral sequences were used to calculate shifts in *pI* across primate evolution and to determine whether the amino acid changes observed in the duplicated howler monkey sequences may have occurred in a common ancestor with other Atelines.

## Data Availability

Sequence reads generated for this study are accessible via the SRA (BioProject accession number PRJNA593273) and assembled sequences have been uploaded as supplemental alignments. Accession numbers for all sequences mined from GenBank are included in Supplementary Table S1. Scripts used for data analyses are available on GitHub (github.com/MareikeJaniak/Alouatta-RNASE1).

## Supporting Information

Supporting Information.
Supporting Information2.
Supporting Information3.
