# Supplementary material for: Duplication and parallel evolution of the pancreatic ribonuclease gene (RNASE1) in folivorous non-colobine primates, the howler monkeys (Alouatta spp.)
Source: Sci Rep. 2019 Dec 30;9:20366. doi: 10.1038/s41598-019-56941-7 (PMC6937293; doi:10.1038/s41598-019-56941-7)
Supplement: Supplementary file 1 — Supporting Information. [file 41598_2019_56941_MOESM1_ESM.pdf]

Duplication and parallel evolution of the pancreatic ribonuclease gene (*RNASE1*) in folivorous non-colobine primates, the howler monkeys (*Alouatta* spp.)

Mareike C. Janiak, Andrew S. Burrell, Joseph D. Orkin, Todd R. Disotell

Supplemental Table S1. NCBI accession numbers for sequences used in this study.

Gene	Species	NCBI Accession Number/Location
RNASE1	*Aotus nancymaae*	NW_012163535.1 [8186684-8187154]
	Ateles geoffroyi	AF449639.1
	Callithrix jacchus	NC_013905.1 [45071412-45071882]
	Cebus capucinus	NW_016107398.1 [302123-302593]
	Chlorocebus aethiops	AF449635.1
	Colobus guereza	DQ516063.1
	Gorilla gorilla	AF449629.1
	Homo sapiens	NC_000014.9 [20801598-20802069]
	Lagothrix lagotricha	AF449640.1
	Lemur catta	AF449641.1
	Macaca mulatta	AF449632.1
	Macaca nemestrina	AF449633.1
	Miopithecus talapoin	AF449636.1
	Nasalis larvatus	DQ494879.1
	Nomascus leucogenys	AF449631.1
	Pan troglodytes	AF449628.1
	Papio hamadryas	AF449634.1
	Pongo pygmaeus	DQ494868.1
	Procolobus badius	DQ494875.1
	Pygathrix nemaeus	AF449642.1
	Rhinopithecus bieti	GQ334693.1
	Saguinus oedipus	AF449638.1
	Saimiri sciureus	AF449637.1
	Tarsius syrichta	NW_007023513.1 [17837-18307]
	Bos taurus	NC_037337.1 [26356008-26357591]
	Alouatta palliata	PRJNA593273
	Alouatta seniculus	PRJNA593273
	Alouatta sara	PRJNA593273
	Alouatta pigra	PRJNA593273
RNASE1B	*Colobus guereza*	DQ516064.1
	Nasalis larvatus	DQ494863.1
	Pygathrix nemaeus	AF449643.1
	Procolobus badius	DQ494873.1
	Rhinopithecus bieti	GQ334696.1
	Alouatta palliata	PRJNA593273
	Alouatta seniculus	PRJNA593273
	Alouatta sara	PRJNA593273
	Alouatta pigra	PRJNA593273
RNASE1C	*Colobus guereza*	DQ516065.1
	Procolobus badius	DQ494874.1
LYZ	*Alouatta palliata*	supplemental alignment
	Callithrix jacchus	U76923.1
	Colobus guereza	U76916.1
	Nasalis larvatus	AH004928.2
	Papio anubis	U76919.1
	Saguinus oedipus	U76922.1
	Saimiri boliviensis	NW_003943629.1 [1119538-1124395]
LYZ2	*Bos taurus*	NM_180999.1

Supplemental Table S2. Primers used for *RNASE1* amplification.

Primer	Sequence	Settings
RNASE1_Alouatta_F	TCCTCCACAGAAGCATCGAA	3 min at 98ºC; 30 cycles of 20 sec at 98ºC, 30 sec at 55ºC, and 30 sec at 72ºC; 5 min at 72ºC
RNASE1_Alouatta_R	AGGTGGTCTCAGGTGATTCT	

Supplemental Table S3. Pairwise identity (%) of lysozyme C (*LYZ*) amino acid sequences

	Bos taurus LYZ2C	*Colobus guereza LYZ*	*Nasalis larvatus LYZ*	*Papio anubis LYZ*	*Alouatta palliata LYZ*	*Callithrix jacchus LYZ*	*Saguinus oedipus LYZ*
Colobus guereza LYZ	75.00						
Nasalis larvatus LYZ	75.00	95.95					
Papio anubis LYZ	68.92	91.22	91.22				
Alouatta palliata LYZ	68.92	84.46	83.78	86.49			
Callithrix jacchus LYZ	68.92	86.49	84.46	89.86	91.89		
Saguinus oedipus LYZ	68.24	85.81	83.78	89.19	91.22	97.97	
Saimiri boliviensis LYZ	68.92	86.49	84.46	89.86	91.22	97.97	97.30

Supplemental Table S4. Variant calls for reads mapped against species-specific *RNASE1* consensus sequence. Bolded positions are common to all species.

Species	Position	Reference allele	Alternative allele(s)	Percentage of reads	
				Ref	Alt
Alouatta palliata (10X)	709	A	G	76.92	17.95
	715	C	T	37.84	59.46
	736	CCTGGTGCTGCTGGTGCTGG	CCTGGTGCTGG	45.45	48.48
	**789-790**	AG	GA	57.45	38.30
	793	C	T	71.74	28.26
	**794**	G	A	57.45	42.55
	830	T	G	70.21	29.79
	**835**	TAGCAGCA	TAGCAGCAGCA	58.14	41.86
	845	C	T	53.33	46.67
	**847**	T	C	52.17	47.83
	859	C	T	80.39	19.61
	862	C	T	71.15	28.85
	**893**	C	T	57.89	42.11
	949	C	T	69.49	30.51
	986	T	G	39.62	58.49
	1027	C	A	82.69	17.31
Alouatta palliata (MiSeq, Apal_S1)	709	A	G	74.64	23.91
	715	C	T	49.13	49.12
	736	CCTGGTGCTGCTGGTGCTGG	CCTGGTGCTGG	40.99	33.74
	**789-790**	GA	AG	50.56	43.16
	793-**794**	CA	TG, CG	53.74	21, 25
	830	T	G	77.62	21.63
	**835**	TAGCAGCA	TAGCAGCAGCA	56.14	29.03
	845	C	T	62.18	37.17
	**847**	T	C	56.00	41.91
	859	C	T	74.87	23.19
	862	C	T	75.79	22.34
	893	C	T	50.21	47.93
	919	G	A	74.40	24.30
	949	C	T	74.90	24.36
	986	T	G	51.29	47.72
	1027	C	A	74.07	25.23
	1129	T	C	72.62	26.51
Alouatta seniculus (Asen_S2)	**789-790**	AG	GA	54.36	38.84
	**794**	G	A	54.54	43.32
	**835**	TAGCAGCA	TAGCAGCAGCA	56.93	30.26
	**847**	T	C	60.62	38.59

	860	A	G	54.90	43.31
	**893**	C	T	51.55	47.50
	986	G	A, T	49.09	48.76
	1041	G	A	50.91	23.70
	1111	C	A	74.24	23.82
Alouatta sara (Bhow_S3)	709	G	A	50.06	49.37
	711	C	T	53.70	44.97
	716	G	A	78.10	21.40
	743	C	T	54.06	40.68
	**789-790**	GA	AG	75.29	22.95
	**794**	A	G	49.43	43.14
	**835**	TAGCAGCA	TAGCAGCAGCA	54.13	43.70
	845	C	T	56.72	29.73
	**847**	T	C	62.51	36.82
	860	A	G	55.87	42.15
	**893**	C	T	53.18	45.99
	1041	G	A	50.60	47.64
	1111	C	A	72.77	25.35
Alouatta pigra (Ghow_S4)	709	G	A	46.39	52.97
	716	G	A	65.85	32.77
	**789-790**	GA	AG	65.91	28.89
	**794**	A	G	39.01	55.14
	830	T	G	42.07	55.87
	**835**	TAGCAGCA	TAGCAGCAGCA	43.67	55.61
	845	C	T	46.89	41.30
	**847**	T	C	47.42	51.69
	**893**	C	T	40.35	57.98
	902	C	T	37.26	60.84
	1027	C	A	39.35	58.62
	1032	G	A	38.57	60.96
	1041	A	G	69.99	26.73
	1071	G	A	40.49	57.85
	1084	G	A	36.81	62.27

Supplemental Table S5. Pairwise identities (%) of *Alouatta RNASE1* haplotype nucleotide sequences.

	Ghow_Hap3	Ghow_Hap2	Bhow_Hap_1	Asen_Hap3	Asen_Hap1	Apal_Hap_1	Apal10X_Hap2	Apal_Hap3	Apal10X_Hap3	Ghow_Hap1	Bhow_Hap_3	Bhow_Hap_2	Asen_Hap2	Apal_Hap_2
**Ghow_Hap3**														
**Ghow_Hap2**	99.57													
**Bhow_Hap1**	98.28	98.71												
**Asen_Hap3**	98.28	98.71	100											
**Asen_Hap1**	98.28	98.28	99.57	99.57										
**Apal_Hap_1**	98.06	98.06	98.49	98.49	98.49									
**Apal10X_Hap2**	98.06	98.06	98.92	98.92	98.92	99.14								
**Apal_Hap3**	98.06	98.06	98.92	98.92	98.92	98.28	99.14							
**Apal10X_Hap3**	98.06	98.06	98.92	98.92	98.92	98.28	99.14	100						
**Ghow_Hap1**	96.34	96.34	96.77	96.77	96.77	96.55	96.98	96.98	96.98					
**Bhow_Hap_3**	96.12	96.12	96.98	96.98	96.98	96.34	96.77	97.2	97.2	99.35				
**Bhow_Hap_2**	95.47	95.91	96.77	96.77	96.34	95.69	96.12	96.55	96.55	98.7	99.35			
**Asen_Hap2**	96.12	96.55	97.41	97.41	97.2	96.34	96.77	96.77	96.77	98.48	99.13	98.92		
**Apal_Hap_2**	93.53	93.53	94.4	94.4	94.61	94.18	95.04	95.04	95.04	96.75	96.53	96.1	96.53	
**Apal10X_Hap1**	93.53	93.53	94.4	94.4	94.61	94.18	95.04	95.04	95.04	96.75	96.53	96.1	96.53	100

Supplemental Table S6. Isoelectric point (*pI*) for all primate *RNASE1* sequences and duplicated sequences, including reconstructed ancestral sequences. *PI* calculated with ExPASy.

Sequence	*pI*
LEMUR_CATTA_RNASE1	8.82
SAGUINUS_OEDIPUS_RNASE1	8.82
CALLITHRIX_JACCHUS_RNASE1	9.10
N7	8.65
AOTUS_NANCYMAAE_RNASE1	7.56
N6	8.43
SAIMIRI_SCIUREUS_RNASE1	8.65
CEBUS_CAPUCINUS_RNASE1	8.81
N8	8.81
N5	8.81
APAL_HAP2	8.43
GHOW_HAP1	8.12
N12	8.43
BHOW_HAP2	8.64
ASEN_HAP2	8.64
N13	8.64
N11	8.43
APAL_HAP1	6.50
GHOW_HAP2	5.81
ASEN_HAP1	6.49
BHOW_HAP1	6.49
N16	6.49
N15	6.50
N14	6.50
N10	8.43
ATELES_GEOFFROYI_RNASE1	8.43
LAGOTHRIX_LAGOTRICHA_RNASE1	8.12
N17	8.43
N9	8.43
N4	8.43
PAPIO_HAMADRYAS_RNASE1	9.26
MACACA_MULATTA_RNASE1	9.16
MACACA_NEMESTRINA_RNASE1	9.15
N22	9.34
N21	9.34
CHLOROCEBUS_AETHIOPS_RNASE1	9.26
MIOPITHECUS_TALAPOIN_RNASE1	9.26
N23	9.34
N20	9.34
RHINOPITHECUS_BIETI_RNASE1	9.22
RHINOPITHECUS_BIETI_RNASE1B	8.43
N27	9.22

PYGATHRIX_NEMAEUS_RNASE1	9.20
PYGATHRIX_NEMAEUS_RNASE1B	6.93
N28	9.20
N26	9.22
NASALIS_LARVATUS_RNASE1	9.22
NASALIS_LARVATUS_RNASE1B	6.93
N29	9.22
N25	9.22
PROCOLOBUS_BADIUS_RNASE1	9.24
PROCOLOBUS_BADIUS_RNASE1B	6.40
PROCOLOBUS_BADIUS_RNASE1C	6.04
N32	6.40
N31	9.24
COLOBUS_GUEREZA_RNASE1	9.11
COLOBUS_GUEREZA_RNASE1B	7.58
COLOBUS_GUEREZA_RNASE1C	7.55
N34	8.43
N33	9.24
N30	9.24
N24	9.24
N19	9.11
NOMASCUS_LEUCOGENYS_RNASE1	8.96
GORILLA_GORILLA_RNASE1	8.96
PAN_TROGLODYTES_RNASE1	9.10
HOMO_SAPIENS_RNASE1	9.10
N38	8.96
N37	8.96
PONGO_PYGMAEUS_RNASE1	9.10
N36	8.96
N35	8.96
N18	8.96
N3	8.65
TARSIUS_SYRICHTA_RNASE1	8.66
N2	8.82
N1	8.82

Supplemental Figure S7. Results of *RNASE1* amplicon sequencing with an Illumina MiSeq Micro V2 cartridge. Number of reads generated for each sample and number of reads mapped to *RNASE1* reference. The percentage of reads that mapped is indicated above each bar.

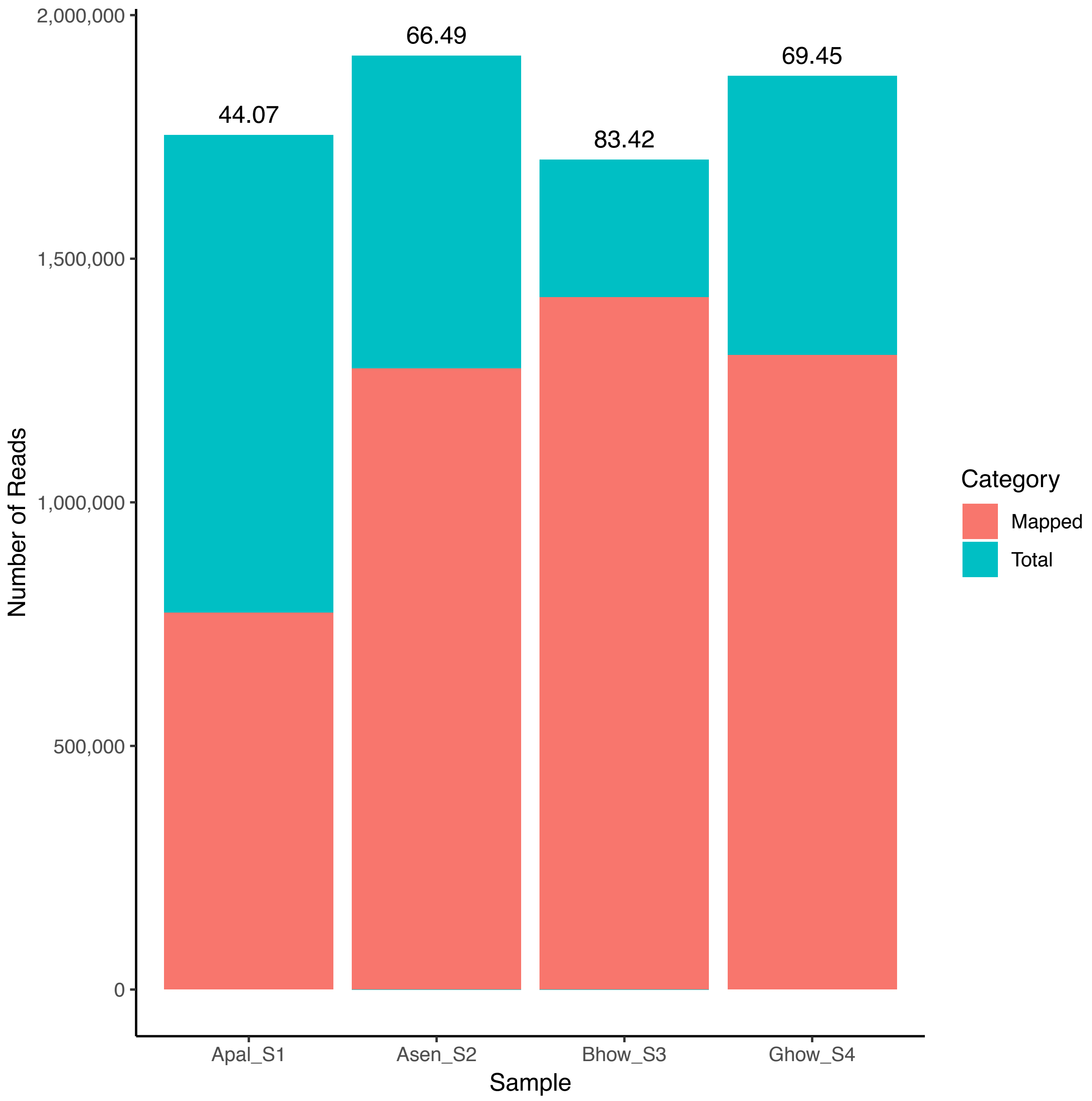

Supplemental Figure S8. Full amino acid alignment of all RNase1 sequences in this study (n = 48). Amino acids identified by previous studies[1,2,3,4] to be of functional importance for the protein's charge, optimal pH, or catalytic efficiency again double-stranded RNA are marked with an asterisk (colobines) or underlined (cow). Corresponding nucleotide sequences are available as a supplemental file.

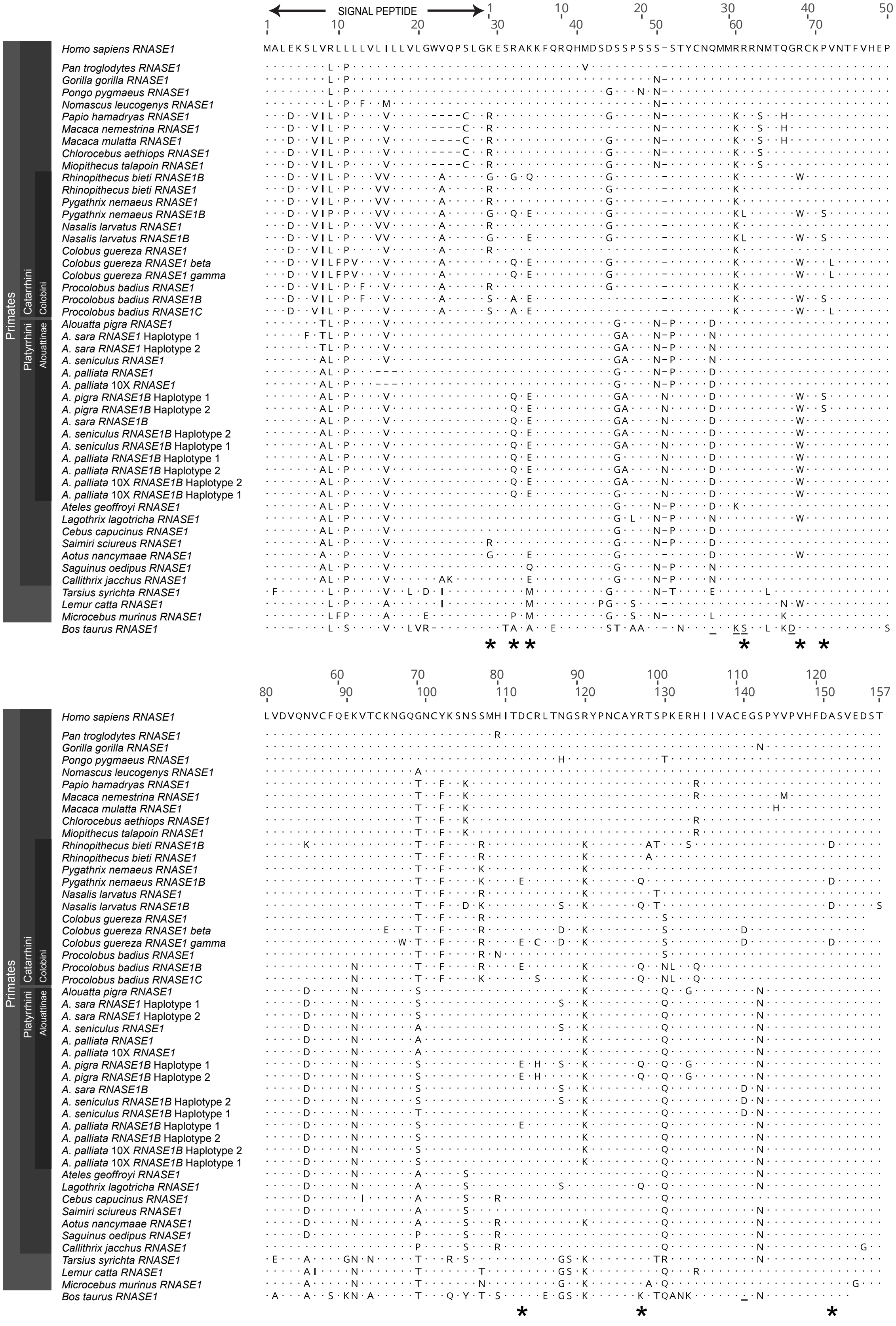

References

1. Jermann, T. M., Opitz, J. G., Stackhouse, J. & Benner, S. A. Reconstructing the evolutionary history of the artiodactyl ribonuclease superfamily. *Nature* **374**, 57–59 (1995)

2. Opitz, J. G. *et al.* Origin of the catalytic activity of bovine seminal ribonuclease against double-stranded RNA. *Biochemistry* **37**, 4023–4033 (1998)

3. Zhang, J., Zhang, Y.-P. & Rosenberg, H. F. Adaptive evolution of a duplicated pancreatic ribonuclease gene in a leaf-eating monkey. *Nat. Genet.* **30**, 411–415 (2002)

4. Zhang, J. Parallel Functional Changes in the Digestive RNases of Ruminants and Colobines by Divergent Amino Acid Substitutions. *Mol. Biol. Evol.* **20**, 1310–1317 (2003)
